# Design of Chitosan-Coated, Quercetin-Loaded PLGA Nanoparticles for Enhanced PSMA-Specific Activity on LnCap Prostate Cancer Cells

**DOI:** 10.3390/biomedicines11041201

**Published:** 2023-04-18

**Authors:** Divesha Essa, Pierre P. D. Kondiah, Pradeep Kumar, Yahya E. Choonara

**Affiliations:** Wits Advanced Drug Delivery Platform Research Unit, Department of Pharmacy and Pharmacology, School of Therapeutic Sciences, Faculty of Health Sciences, University of the Witwatersrand, 7 York Road, Parktown, Johannesburg 2193, South Africa

**Keywords:** PLGA nanoparticles, chitosan, folic acid, prostate-specific membrane antigen, quercetin, active targeting

## Abstract

Nanoparticles are designed to entrap drugs at a high concentration, escape clearance by the immune system, be selectively taken up by cancer cells, and release bioactives in a rate-modulated manner. In this study, quercetin-loaded PLGA nanoparticles were prepared and optimized to determine whether coating with chitosan would increase the cellular uptake of the nanoparticles and if the targeting ability of folic acid as a ligand can provide selective toxicity and enhanced uptake in model LnCap prostate cancer cells, which express high levels of the receptor prostate-specific membrane antigen (PSMA), compared to PC-3 cells, that have relatively low PSMA expression. A design of experiments approach was used to optimize the PLGA nanoparticles to have the maximum quercetin loading, optimal cationic charge, and folic acid coating. We examined the in vitro release of quercetin and comparative cytotoxicity and cellular uptake of the optimized PLGA nanoparticles and revealed that the targeted nano-system provided sustained, pH-dependent quercetin release, and higher cytotoxicity and cellular uptake, compared to the non-targeted nano-system on LnCap cells. There was no significant difference in the cytotoxicity or cellular uptake between the targeted and non-targeted nano-systems on PC-3 cells (featured by low levels of PSMA), pointing to a PSMA-specific mechanism of action of the targeted nano-system. The findings suggest that the nano-system can be used as an efficient nanocarrier for the targeted delivery and release of quercetin (and other similar chemotherapeutics) against prostate cancer cells.

## 1. Introduction

Globally, prostate cancer is a major cause of male mortality, associated with 7% of male cancer-related deaths and 14% of cancer diagnoses [1]. Current therapy includes surgery, radiation, and conventional chemotherapy, with each route resulting in a range of undesirable side effects [2], highlighting the need for alternative treatment options. The advantages of nanotechnology in designing advanced drug delivery systems have been explored extensively since the first nano-formulation. Doxil, a liposomal system carrying the cancer drug doxorubicin, was clinically approved for cancer treatment in 1995 [3]. Nanotechnology in medicine has been used as a tool to minimize off-target losses of the active pharmaceutical ingredient (API) [4,5], improve biodistribution [6], and minimize side effects and systemic exposure [7], hereby enhancing the overall efficacy, safety, and pharmacokinetic and pharmacodynamic profile of the API [8]. Nanoparticle-based treatments achieve this by the precision engineering of their components to improve site-specific targeting and penetration and to increase the solubility and bioavailability of the payload [9].

Quercetin is a plant-derived flavonoid that is found abundantly in fruits and vegetables. It has numerous medicinal properties, including anticancer activity, but its application as an anticancer drug is hampered by poor aqueous solubility, low bioavailability, and chemical instability [10,11]. Therefore, it is an ideal candidate as the API in a nanoparticle-based system, and such studies have been conducted and tested on several different cancers, including prostate cancer [7,8]. Additionally, its solubility and bioavailability limitations are characteristic of many cancer drugs [12,13,14] and, therefore, can also be used as a model therapeutic in preliminary studies for more toxic chemotherapeutic drugs.

Chitosan, a natural polymer derived from the shells of crustaceans, has been widely used for drug delivery applications because of its high biocompatibility. It has a strong positive charge in solution, which can increase cancer cell uptake through interactions with the negatively charged cell membrane [15]. There are also several studies on chitosan nanoparticle systems that demonstrate its ability to release its payload more efficiently at an acidic pH [16,17,18], which is relevant for cancer treatment as the tumor microenvironment is reported to lie in the acidic pH range of 5.6–6.8 [19,20].

The “magic bullet” effect is a term coined by Nobel prizewinner Paul Ehrlich in 1909 [21], describing the ability of a nano-system to target cancer cells for therapeutic effect while causing relatively no harm to surrounding healthy tissue. This can include active targeting of the cancer cells, which involves attaching a targeting ligand to the nanoparticle surface to specifically bind receptors highly expressed on cancer cells but not on healthy cells. Thereafter, the cancer cell receptors allow only the targeted nanoparticles to enter the cell, where they can release the API [22]. Prostate-specific membrane antigen (PSMA) is a cell surface receptor that expresses highly on prostate cancer cells but minimally on non-malignant cells. This makes it an excellent biomarker for prostate cancer, and various reports detail the use of antibodies, aptamers, and small molecules attached to nanoparticles in order to target PSMA on prostate cancer cells [23]. Folic acid is well known for its action as a targeting agent for folate cell surface receptors, and various reports demonstrate its targeting efficiency in nano-systems [24,25,26]. We used it as a targeting ligand to expand on the limited studies detailing its binding ability for PSMA [22,23], choosing LnCap and PC-3 as PSMA-positive and -negative cell lines, respectively. Importantly, both cell lines do not express high levels of folate [22], eliminating the possibility of competitive binding for folate. Therefore, the folic acid moiety in our system was used to specifically bind to PSMA, to allow for the nanoparticles to preferentially enter LnCap cells via receptor-mediated endocytosis, wherein the entrapped payload could be released in a controlled manner [27]. 

**Figure 1:**
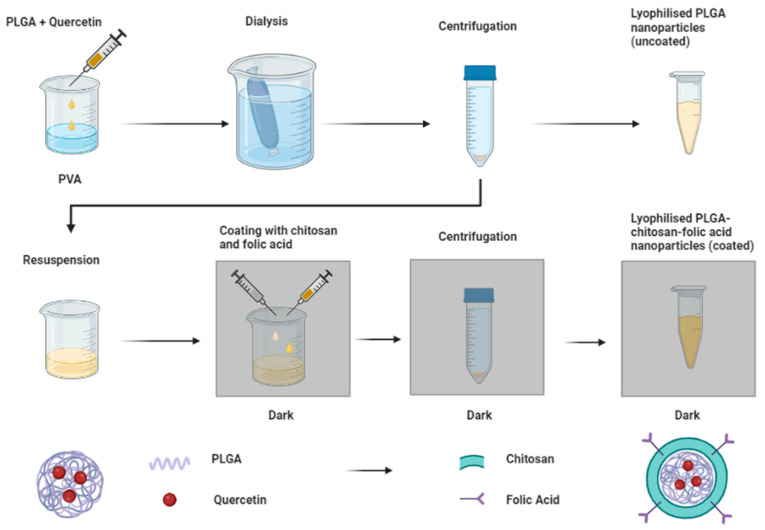
Fabrication process of uncoated (PLGA) and coated (PLGA-chitosan folic acid) nanoparticles.

Our system included poly(lactic-*co*-glycolic) acid (PLGA), which is a biodegradable, biocompatible FDA-approved polymer. PLGA is widely used in drug delivery systems and is useful in our system of nanoparticle formation with the hydrophobic quercetin component, as it forms non-polar interactions with the molecule and stabilizes the matrix by maintaining quercetin in the core of the nanoparticle, separate from the surrounding aqueous medium [24,28,29,30]. Here, PLGA was used to entrap quercetin, forming nanoparticles, which were then coated with chitosan and folic acid, as shown in Figure 1. 

Quercetin-entrapped nanoparticles have been previously prepared with PLGA alone [31], in combination with PLGA and folic acid [32], and with chitosan [33] but not with all three components. Nano-systems prepared for drug delivery have been focused on highly positive (cationic) or highly negative (anionic) surface charge for formulation stability and increased interaction with cell membranes [34] even though nanoparticles that are too positively charged could damage the cell membrane and anionic particles might be repelled by the cell membrane, which is also negatively charged. Furthermore, highly charged nanoparticles are rapidly cleared by the immune system, while more neutral nanoparticles exhibit longer clearance times [6]. Therefore, we chose to investigate the activity of slightly positively charged nanoparticles, which, as far as we are aware, there are no data about. 

Design of experiments is a tool used in formulation science to analyze the data obtained from a set of experiments and make predictions on how to tailor the fabrication process to formulate a product with the most desired properties [35]. We aimed to use this methodology to fabricate nanoparticles optimized for the highest loading of quercetin, a positive surface charge as close to neutral as possible, and the highest folic acid content to maximize targeting efficiency. 

## 2. Materials and Methods

### 2.1. Materials 

Poly(D,L-lactide-co-glycolide)(PLGA) (lactide/glycolide ratio of 50:50, molecular weight 7000–17,000 with acid end groups, low molecular weight chitosan, anhydrous dimethyl sulfoxide (DMSO), folic acid, sodium hydroxide pellets (NaOH), glacial acetic acid, polyvinyl alcohol (MW 23,000–28,000) (PVA), quercetin hydrate, dialysis tubing (MWCO 14 KDa), sodium carbonate, methanol, PBS buffer tablets (pH 7.4), fluorescein iso-thiocyanate (FITC), and tween80 were purchased from Merck (Pty) Ltd., Estate South, Modderfontein, Gauteng, South Africa. 

### 2.2. Preparation of Nanoparticles

Nanoparticles were prepared by the nanoprecipitation method, modified from [36], where PLGA was dissolved in 1 mL of DMSO and added dropwise under stirring to the aqueous phase, consisting of 0.5% PVA. For the quercetin- and FITC-loaded nano-systems, quercetin or FITC were also dissolved in the organic phase before the addition. The solution was then dialyzed for 24 h to remove DMSO, and the PVA was subsequently removed by centrifugation for 30 min at 12,000 rpm (3 times). These nanoparticles were designated uncoated. For the “coated” nanoparticles, the centrifugal pellet was resuspended and incubated in the dark, at room temperature, for 1.5 h, with solutions of chitosan and folic acid. The chitosan solutions were prepared by dissolving chitosan in 1% glacial acetic acid and were subsequently filtered, while the folic acid solutions were prepared by dissolving folic acid in 0.4 M NaOH [37]. After the incubation period, the nanoparticles were separated by centrifugation and frozen at −80 °C for 24 h. Frozen samples were then lyophilized (Freezone 12 lyophilizer, Labcono, Kansas City, MO, USA) for 24 h. Henceforth, the PLGA nanoparticles are referred to as “uncoated nps”, and the PLGA/chitosan folic acid nanoparticles are referred to as “coated nps”.

#### Optimization by Design of Experiments

For the optimization of the nanoparticles, design of experiments was implemented, where the mass of quercetin, mass of chitosan and mass of folic acid were chosen as the independent variables while the zeta potential, quercetin loading and folic acid content were measured as the dependent response variables. Using JMP 17 software, a face central composite design, with two center points was used to generate 16 formulations, listed in Table 1. These formulations were then prepared by the above method.

### 2.3. Particle Size, Polydispersity Index, and Zeta Potential Analysis Using Dynamic Light Scattering

The nanoparticles were dissolved (1 mg in 1 mL of distilled water) and analyzed using dynamic light scattering (DLS) on a ZetaSizer NanoZS (Malvern Instruments Ltd., Worcestershire, UK) particle size analyzer. Samples were diluted with distilled water before measurement in capillary cells (Malvern Instruments Ltd., Malvern, Worcestershire, UK). The temperature of the samples was maintained at 25 °C throughout the analyses.

### 2.4. Determination of Folic Acid Content by Ultra-Violet (UV) Spectrophotometry

The folic acid content was determined by a method modified from [38]. Briefly, 5 mg of nanoparticles were dispersed in 1 mL of 0.2 M sodium carbonate and vortexed for 1 min. The dispersions were then centrifuged at 3500 rpm for 15 min, and the absorbance of the supernatants was read at 283 nm using the Nanophotometer UV/Vis spectrophotometer NP80 (Implen, Munich, Germany). The folic acid content was assayed against standards of known concentrations of folic acid dissolved in 0.2 M sodium carbonate and was used to determine the percentage of folic acid in the nanoparticle formulations.

### 2.5. Characterization Using Fourier Transform Infra-Red (FTIR)

The starting materials and nanoparticle systems were subjected to FTIR analysis, and characteristic peaks were compared to confirm the structure of the pristine polymers and the nano-systems. The spectra were recorded using a PerkinElmer Inc. (Waltham, MA, USA) spectrometer with a single reflection diamond MIRTGS detector. Samples were processed by a universal attenuated total reflectance (ATR) polarization accessory, at a resolution of 4 cm^−1^, with a constant pressure of 110 psi. 

### 2.6. Investigation of Thermal Degradation by Thermogravimetric Analysis (TGA)

The temperature ranges with which the samples degrade was studied using a thermogravimetric analyzer (TGA) (PerkinElmer, TGA 4000, Llantrisant, Wales, UK). Starting materials and samples were allowed to reach 30 °C and then heated at a rate of 10 °C min^−1^ to 800 °C. An inert environment was maintained for the samples by constant purging of nitrogen gas for the duration of the run.

### 2.7. Surface and Crystallinity Experiments Using Powder X-Ray Diffraction (XRD) 

The lyophilized samples and pristine polymers were crushed to form fine powders, which were then loaded and smoothed onto a sample holder for analysis. X-ray diffraction spectra were generated on a benchtop MiniFlex 600 (Rigaku, Tokyo, Japan) powder diffractometer. CuKα radiation at 40 kV and 15 mA were set as the parameters for all experiments. Data were recorded using a 2θ scan range of 10–60 degrees at a scan rate of 10° min^−1^. These powder X-ray diffraction analyses indicate the degree of crystallinity and amorphous nature of the polymers and nanoparticles and hence provide information about their surface properties and behavior.

### 2.8. Phase Transition Studies Employing Differential Scanning Calorimetry (DSC)

The thermal properties of the loaded and unloaded nano-systems and starting materials were investigated using a differential scanning calorimeter (DSC) (Mettler Toledo, DSC, STAReSystem, Schwerzenbach, ZH, Switzerland). DSC measurements provide information about the thermal and phase changes of the samples and are used in this case to compare how these properties change once quercetin is loaded into the nano-system. The nanoparticle and quercetin samples of ~5 mg were weighed into aluminum crucibles, which were sealed and then heated over a temperature range of 0 to 400 °C at a heating rate of 10 °C min^−1^. The samples were maintained in an inert N_2_ gas atmosphere. 

### 2.9. Scanning Electron Microscopy (SEM) of Sample Suspensions

Suspensions of lyophilized samples in distilled water were diluted, dropped onto aluminum stubs, and dried for 48 h under vacuum. In order to induce electrical conduction, samples were coated with a fine layer of gold under vacuum, using a sputter coater. Coated samples were analyzed on a ZEISS SIGMA 03-39 Field Emission Scanning Electron Microscope at 5–15 kV acceleration voltage under an argon atmosphere.

### 2.10. UV Spectrophotometric Analysis of Quercetin Loading in Nanoparticle Systems

Nanoparticles were dispersed in PBS (pH 7.4) at a concentration of 10 mg/mL and incubated at 37 °C for 2 h using an Orbit shaker incubator (LM-530-2, MRC Laboratory Instruments Ltd., Hahistradrut, Holon, Israel) at 50 rpm. Thereafter, 50 µL of the solution was added to 50 µL of DMSO and 900 µL of methanol, a method modified from [36]. The mixture was vortexed for 30 s and centrifuged at 14,000 rpm for 5 min, and the absorbance of the supernatant read at 371 nm [39]. Quercetin standards in methanol were used to construct a calibration curve, and samples were analyzed using the Nanophotometer UV/Vis spectrophotometer NP80 (Implen, Munich, Germany). Quercetin loading was represented as the percentage of quercetin per mg of nanoparticles.

### 2.11. In Vitro Release of Quercetin

In vitro release studies of the nanoparticle systems were performed in phosphate-buffered saline (PBS) at pH 7.4 at 37 °C to simulate physiological pH and at pH 6.0 to simulate the pH of the tumor microenvironment [19]. The method was adapted from [40]. A total of 10 mg of nanoparticles was suspended in 2 mL of PBS in a dialysis bag placed in 50 mL of PBS containing 0.5% (*v*/*v*) tween 80 and stirred for 72 h at 37 °C. Samples were prepared in triplicate and compared with unloaded nanoparticles. At specific time points, 0.5 mL of the release medium was withdrawn and replaced with an equal volume of the fresh medium. Samples were diluted and centrifuged, and quercetin concentration was analyzed spectrophotometrically at 371 nm. 

### 2.12. Cell Culture Conditions and Cytotoxicity Studies

3T3-NIH mouse fibroblast cells and PC-3 and LnCap prostate carcinoma cells were obtained from Cellonex (Johannesburg, South Africa). Cells were confirmed to be free of mycoplasma. 3T3-NIH and PC-3 cells were grown in the RPMI culture medium, and LnCap cells were grown in DMEM/F12 culture medium. The media were supplemented with 10% FBS and 1% penicillin/streptomycin as per cell culture protocols. 3T3-NIH, LnCap, and PC-3 cells were used at passage numbers 5–11. When confluent, PC-3 and LnCap cells were seeded in 96-well plates at a cell density of 5 × 10^3^ cells/well, and post-cell attachment was treated with quercetin and quercetin-loaded nanoparticles. 3T3-NIH cells were seeded at a density of 3 × 10^3^ cells/well and treated with unloaded nanoparticles as a toxicity control. A total of 72 h after treatment, cell viability was evaluated using the (3-(4,5-dimethylthiazol-2-yl)-2,5-diphenyltetrazolium bromide) (MTT) assay.

### 2.13. Cellular Uptake of Nanoparticles

PC-3 and LnCap cells were seeded onto coverslips in 6-well plates at a cell density of 1 × 10^5^ cells/well and treated with FITC-loaded nanoparticles. A total of 24 h post-treatment, cells were washed several times with PBS, fixed with 4% paraformaldehyde, and stained with DAPI. The coverslips were then mounted onto glass slides for fluorescence microscopy using an Olympus BX41 Fluorescence Microscope (Olympus Corporation, Tokyo, Japan). The fluorescence intensity of images was calculated using ImageJ software, version 1.54.

### 2.14. Statistical Analysis

Measured data were calculated as the average of three experiments and represented with the standard error of the mean. Data between different experimental groups were compared using one-way analysis of variance (ANOVA) on Origin V8.5 software. *p* values of less than 0.05 were considered significant.

## 3. Results

### 3.1. Optimized Formulation Results Using DOE

#### 3.1.1. Quercetin Loading, Size, and Potential and Folic Acid Conjugation of Formulations

There was a size increase after coating nanoparticles with chitosan and folic acid (Table 4). A response surface design was used to create a quadratic model for the formulations and the order of experiments listed in Table 2. The limits of quercetin, chitosan, and folic acid were based on their solubility and previous studies [37,41,42].

#### 3.1.2. Analysis of Responses

**Figure 2:**
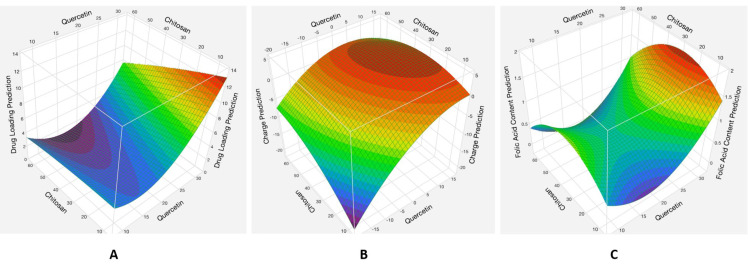
Response surface plots showing quercetin loading (**A**), charge (**B**), and folic acid content (**C**), variation with different quercetin and chitosan amounts. Amount of folic acid was kept constant at the intermediate value of 30 mg. Obtained from JMP V17.

The analysis showed that the quercetin and chitosan quadratic factors and the interaction between the amount of quercetin and the amount of chitosan had significant effects (*p* < 0.05) on all the measured responses, while the amount of folic acid did not have a significant effect (*p* > 0.05) on the responses (Figure S3). The data were shown to fit the quadratic model with R^2^ values between 0.85 and 0.97 (Figure S1) with a normal distribution (Figure S2). The interaction between the amounts of quercetin and chitosan and its effect on quercetin loading, surface charge, and folic acid conjugation is shown in Figure 2. 

##### Effects of the Interaction between the Quercetin and Chitosan Factors

When both quercetin and chitosan are low, there are intermediate quercetin loading values, and when quercetin is high and chitosan is low, there are high quercetin loading values. This is because of the higher total amount of quercetin in the formulation up until the point where the maximum amount of quercetin is entrapped, and adding more quercetin does not increase the loading. Increasing the initial amount of chitosan decreases the quercetin loading in an almost linear fashion. This is because of the lower fraction of quercetin in the formulation, as increasing the chitosan amount increases the total amount of product.

The amount of quercetin inversely affects the surface charge of the nanoparticles. This could be because quercetin is negatively charged and therefore reduces the total charge. The amount of chitosan, which is positively charged, increases the surface charge of the nanoparticles up until the maximum amount of chitosan coating that could be achieved by this method. The folic acid conjugation increases with the amount of quercetin in the system. This could be because as quercetin decreases the surface charge of the uncoated nanoparticles, it increases the amount of chitosan coating and therefore increases the amount of negatively charged folic acid molecules that can adsorb to the positive charge. This also explains why increasing the chitosan amount increases folic acid conjugation until the value of 30 mg, which could be at the maximum amount of chitosan coating, and any further increase in chitosan leads to neutralization of the folic acid by the uncoated chitosan in solution.

**Figure 3:**
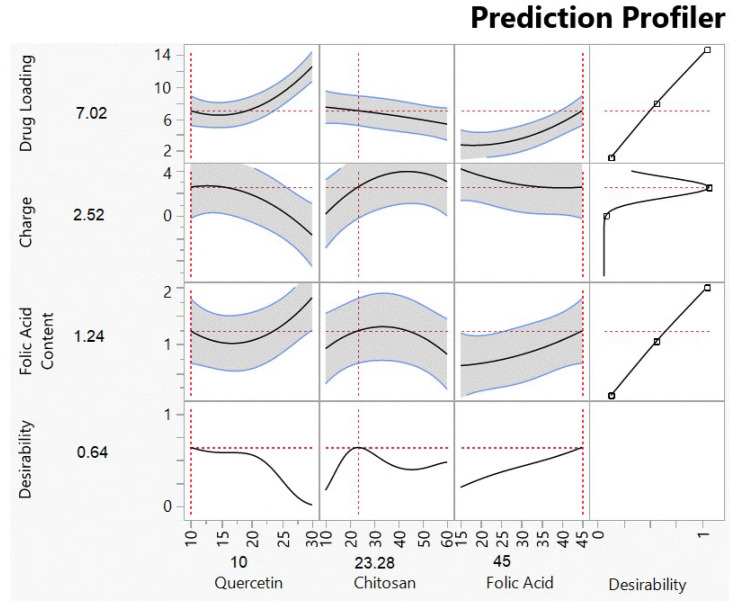
Prediction profiler showing optimized solution with maximum desirability, from JMP V17.

**Figure 4:**
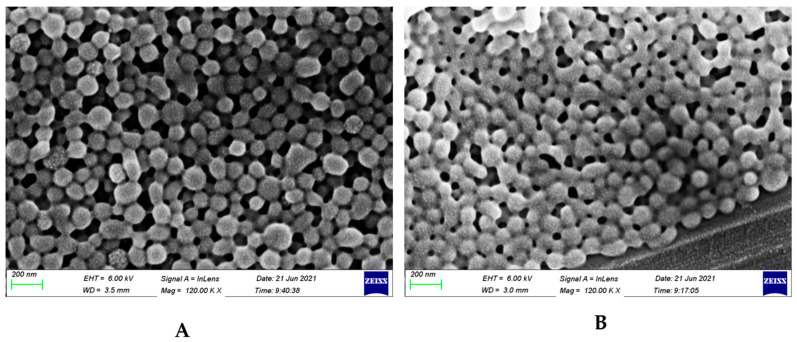
Scanning electron micrographs of (**A**) uncoated nanoparticles and (**B**) coated nanoparticles.

The optimized solution, shown in Figure 3, was the input of 10 mg of quercetin, 23 mg of chitosan, and 45 mg of folic acid. The actual versus predicted responses shown in Table 3 reflects values within the limits of the predicted solution (shown in Figure 4). This formulation was used in all further experiments.

### 3.2. Size and Potential Data Show Spherical Coated Particles with Positive Surface Charge

The particle size diameter of the uncoated nanoparticles increased from 159.8 ± 2.0 to 206.2 ± 1.7 nm upon coating with chitosan and folic acid (Figure S4(A,B-1)) and Table 4. The polydispersity indices were very low (Table 4), suggesting precise, uniform particles. The particle uniformity can also be seen in the scanning electron micrographs (Figure 4), which show spherical particle morphology. The SEM images also suggest slightly smaller diameters (using the scale bar of 200 nm) than the dynamic light scattering measurements in Figure S4(A,B-1), which could be due to the fact that the DLS technique measures the hydrodynamic diameter of the particles in solution whereas scanning electron microscopy examines the particles in solid form, where the hydrated polymeric shell has collapsed during drying and under the vacuum of the SEM chamber [42,43]. Figure 4B also show some adhesion between the individual nanoparticles, which could be due to the interaction between the moieties of the chitosan component of the coating [42]. The zeta potential results show that the surface charge of the uncoated nanoparticles increased from −21.0 ± 1.6 to +1.84 ± 0.4 mV after coating with chitosan and folic acid (Figure S4(A,B-2) and Table 4). The negative charge of the uncoated nanoparticles is due to the carboxylic acid end groups in the PLGA polymer, whereas the small positive charge of the coated nanoparticles is due to the highly positive charge of the amine groups on the chitosan moiety and the negative charge of the acid end groups on the folic acid moiety.

### 3.3. Molecular Structure by FTIR Shows Adsorption Interactions and XRD Show Amorphous Nanoparticle Structure

**Figure 5:**
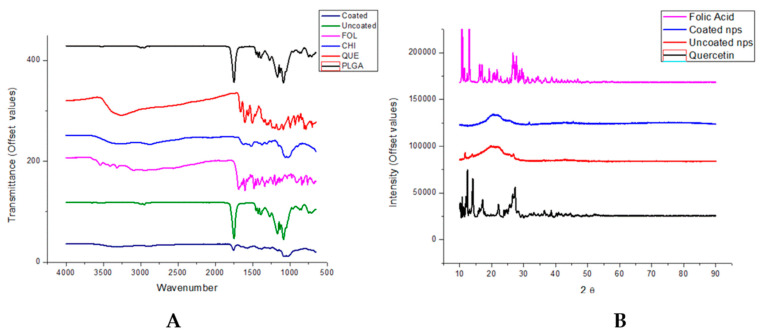
(**A**) FTIR spectra of uncoated and coated nps and pristine polymers where Fol = folic acid, Chi = chitosan, and Que = quercetin. (**B**) XRD spectra of Quercetin and uncoated and coated nanoparticles.

FTIR spectra of all reagents and products are shown in Figure 5A. The PLGA spectrum shows characteristic strong bands at 1750 cm^−1^ and 1094 cm^−1^ due to C=O and C–O–C stretching, respectively, C–H bending at 1200–1400 cm^−1^, and small bands due to linear C–H stretching vibrations at ~2990 and 3000 cm^−1^. In the spectrum of chitosan, bands at ~1110 cm^−1^ can be observed due to amine stretching and bands at 1600–1700 cm^−1^ due to amide bonds. Bands due to C–H bending at 1400 cm^−1^ and C–H stretching at 2900 cm^−1^ can also be observed. The spectrum for folic acid shows a number of bands between 2800 and 3600 cm^−1^ due to N-H and OH stretching, with a sharp band at 1700 cm^−1^ due to C=O amide stretching. A band due to the OH phenyl group is also found at 1400 cm^−1^. These observations were consistent with previous findings [42,43,44,45,46].

The characteristic bands of chitosan can be seen in the spectrum of the coated nanoparticles but not in that of the uncoated nanoparticles, while the strong characteristic PLGA band at 1750 cm^−1^ can be seen in both uncoated and coated nanoparticle spectra. This indicates that the chitosan had been successfully coated onto the surface of the PLGA nanoparticles. The specific bands of folic acid are not clearly visible in the spectra of the coated nanoparticles. This could be due to the larger relative concentrations of the PLGA and chitosan in the nanoparticle system, which could overwhelm the bands due to the folic acid constituent. The spectra of the nanoparticles indicate that the components interact by physical adsorption alone since there are no new bands and, therefore, no formation of new chemical bonds in the nanoparticle systems [37]. 

XRD (Figure 5B) shows the crystallinity of quercetin and folic acid, showing several sharp bands, while the nanoparticles are more amorphous. The spectrum of the uncoated nanoparticles shows more crystallinity, with bands corresponding to those of quercetin, which could be because there is some quercetin at the surface of the nanoparticles. In the spectrum of the coated nanoparticles, these peaks are indistinct and less intense, indicating that quercetin is entrapped more completely within the core of these nanoparticles [47].

### 3.4. Thermal Degradation and Phase Transition Show Retention of Polymer Properties

**Figure 6:**
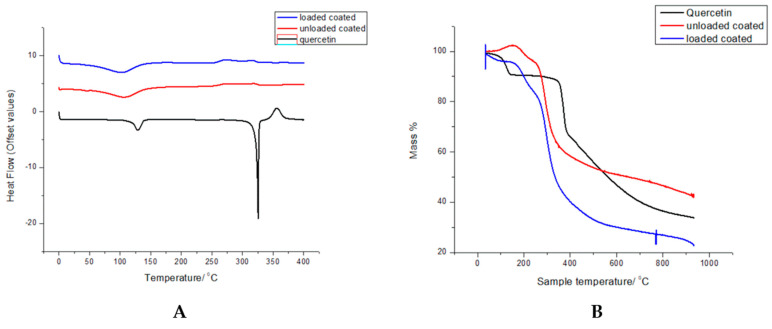
(**A**) DSC and (**B**) TGA thermograms of loaded and unloaded coated nanoparticles where Que = quercetin.

The DSC thermograms are shown in Figure 6A, where native quercetin showed endothermic peaks at 125 and 325 °C, corresponding to its melting and decomposition temperatures, respectively [48]. The thermograms of loaded coated nanoparticles are similar to that of the unloaded nanoparticles, with a broad endothermic peak at 100 °C, corresponding to a characteristic dehydration peak of chitosan [46], but no definite peaks due to melting or degradation. The sharp peaks in the quercetin thermogram are not visible, indicating that quercetin is entrapped within the nanoparticle in an amorphous state [49].

**Figure 7:**
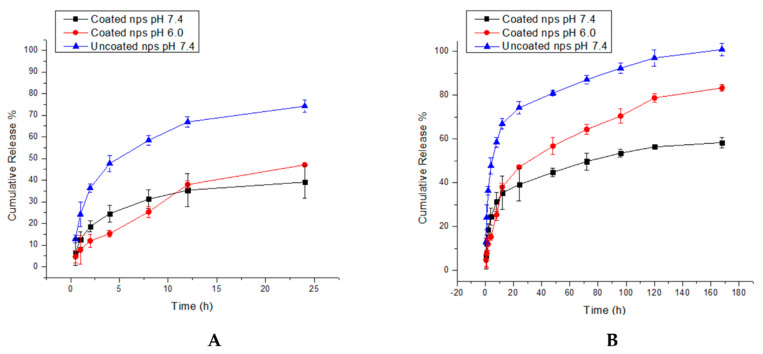
Release curves of quercetin from coated and uncoated nanoparticles (**A**) 24 h and (**B**) 168 h.

For TGA (Figure 6B), weight loss of the nanoparticle systems can be divided into the stages where weight loss is due to the water loss from the chitosan component at 0–300 °C [41] and the thermal degradation stage until 400 °C for the unloaded nanoparticles and 450 °C for the loaded nanoparticles. Quercetin undergoes thermal oxidation at 100–200 °C and, thereafter, degradation at 400 until 800 °C [50]. The weight loss steps observed correspond with the temperatures of the degradation steps observed by the DSC thermograms (Figure 7A). The loaded nanoparticles exhibit greater thermal stability compared to both the unloaded nanoparticles and pristine polymers, probably due to nanoparticle interactions and intact structural conformation, which is consistent with previous studies with the individual components [37,46,47,51].

### 3.5. In Vitro Kinetic Study of Nanoparticle Systems Shows Efficient Quercetin Release

At physiological pH of 7.4, both coated and uncoated nanoparticles displayed a biphasic release pattern, as shown by Figure 7A,B, with an initial burst release followed by a slower release. The uncoated nanoparticles showed a markedly higher burst release, with 48% and 68% release by 4 and 12 h, respectively, compared to 25% and 35% release by the coated nanoparticles at the same time points. The burst release is caused by the adsorption of quercetin molecules on the surface of the nanoparticles, allowing for rapid desorption and diffusion in solution due to the weak interactions between the quercetin and polymer layers at the nanoparticle surface [52,53]. In the case of the coated nanoparticles, the slower burst release suggests strong physical interactions between quercetin and the coating polymer layer, possibly due to the polysaccharidic chitosan component. However, the maximum release at pH 7.4 was only 55% for the coated nanoparticles compared to 98% for the uncoated nanoparticles after 168 h. This could be due to the swelling of the chitosan layer in solution, forming a physical barrier to the diffusion of quercetin out of the nanoparticle core [54].

There is a much shorter burst release by the coated nanoparticles at pH 6.0 (compared to both of the nano-systems tested at pH 7.4) of 12% at 2 h, then a constant release until 36%, where it matches the cumulative release of the coated nanoparticles at 12 H. This could be due to the rapid swelling in nanoparticle size due to the protonation of the amino groups in the chitosan layer, which increases the time taken for the quercetin molecules to diffuse into bulk solution [16]. Thereafter, once the nanoparticles reach equilibrium swelling, the low pH allows for increased solubility of the chitosan layer (since the pH of 6.0 is lower than the pKa of chitosan, which is 6.3), resulting in a faster and more complete release of 78% of the quercetin molecules from the nanoparticle core [40,49]. Importantly, the dissolution of the chitosan layer results in a gradual decrease in the thickness of the chitosan coating around the core, and therefore the coated nanoparticle system displays a more controlled and sustained release profile than that of the uncoated nanoparticles [54].

### 3.6. Increased Cytotoxicity of Quercetin in Nanoparticle System

**Figure 8:**
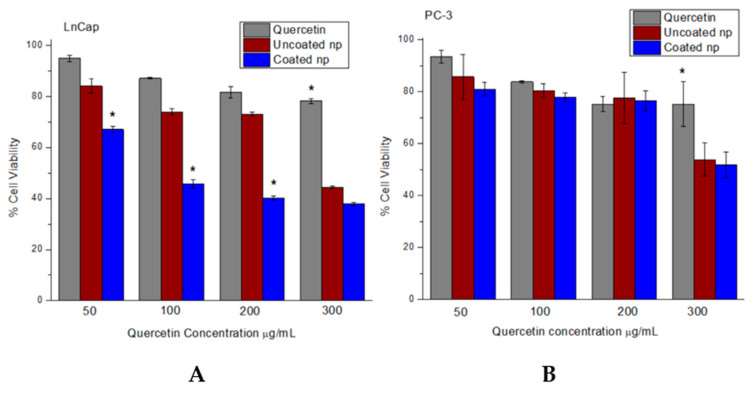
In vitro cell viability of (**A**) LnCap and (**B**) PC-3 cells after being treated for 72 h with free quercetin, uncoated, and coated nanoparticles (* *p*-value < 0.05).

The unloaded nano-systems showed no toxicity on the 3T3-NIH fibroblast cell lines, with viabilities greater than 90% at all concentrations tested (Figure S5). As shown by Figure 8, after 72 h, both cell lines showed a general dose-dependent decrease in viability with increasing concentration of quercetin. The nanoparticle systems showed significantly (*p* < 0.05) more cytotoxicity than free quercetin on the PC-3 cell line (Figure 8B) only at a concentration of 300 µg/mL (50–52% for the nanoparticles vs. 75% for free quercetin). Moreover, at all concentrations, there was no significant difference in the cytotoxicity of the coated and uncoated nanoparticles on this cell line. However, the coated nanoparticles were significantly more cytotoxic than both free quercetin and uncoated nanoparticles on the LnCap cell line at all concentrations except for 300 µg/mL, where the nanoparticle systems were not significantly different from each other, but both were more cytotoxic than free quercetin (38% for the coated nanoparticles vs. 44% for the uncoated nanoparticles and 78% for free quercetin). This corresponds to the concentration that the nanoparticle systems were significantly different from quercetin in the PC-3 cell line (noted above). The coated nanoparticles were also more cytotoxic on the LnCap cell line at lower concentrations than the PC-3 cell line, with the viability of 48%, 40%, and 38% for LnCap compared to 77%, 70%, and 50% for PC-3 at concentrations of 100, 200, and 300 µg/mL, respectively. The selective cytotoxicity of the coated nanoparticles on the LnCap cell line could indicate a greater nanoparticle cellular uptake by this cell line compared to the PC-3 cell line. This could be due to the PSMA receptors on the surface of the LnCap cells (but not on PC-3 cells) that allow the coated nanoparticles to enter the cells through the binding of the folic acid moiety, hereby increasing intra-cellular quercetin concentration and causing inhibition of cell growth [51]. However, the lack of significance of the toxicity difference between the coated and uncoated nano-systems on both cell lines at a concentration of 300 µg/mL indicates that this could be the concentration at which the nanoparticles are able to enter the cells by some mechanism other than active targeting of the PSMA receptor.

### 3.7. Greater Cellular Uptake of Targeted Nanoparticles in a PSMA-Positive Cell Line

**Figure 9:**
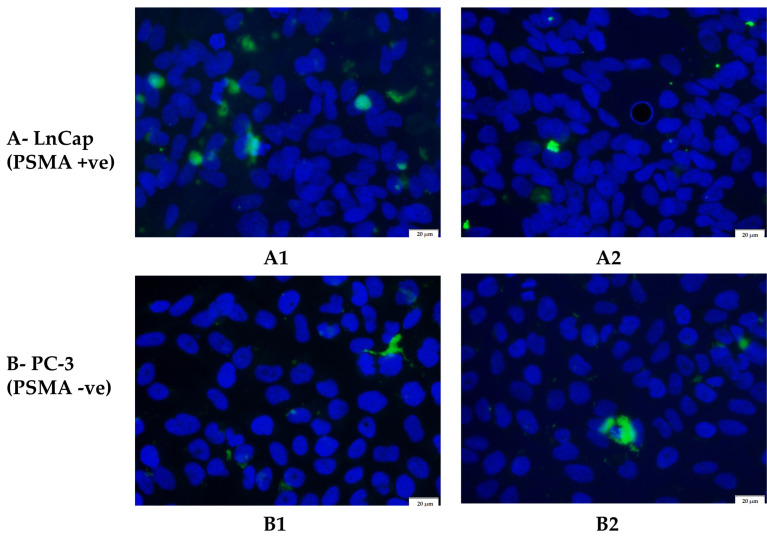
(**A**) LnCap cells and (**B**) PC-3 cells treated with coated (**1**) and uncoated (**2**) nanoparticles.

PSMA-positive LnCap and PSMA-negative PC-3 cells were treated with uncoated and coated nanoparticles that had FITC entrapped within their core. As shown by Figure 9, there is a definite increase in cell association of FITC moieties (shown as green) around the nuclei (shown as blue) in the LnCap cell line when treated with the coated nanoparticles (Figure 9(A1)). There is also more green fluorescence in the LnCap cell line that was treated with uncoated nanoparticles (Figure 9(A2)) compared to the PC-3 cell line treated with either coated or uncoated nanoparticles (Figure 9(B1,B2)). As expected, there is no significant difference in fluorescence between the coated and uncoated nanoparticle treatments on the PC-3 cell line (Figure 9(B1,B2)) and Table 5. This suggests a greater selective uptake of the coated nanoparticles by the PSMA-positive LnCap cell line compared with the PSMA-negative PC-3 cell line, which could be facilitated by the binding of the PSMA receptors on the LnCap cells by the folic acid moieties on the surface of the coated nanoparticles, allowing them to enter the cells by receptor-mediated endocytosis [52,55,56].

## 4. Discussion

We used response surface methodology in JMP V17 with a face-centered cubic design to generate 16 formulation parameter combinations shown in Table 1. This design was selected as the solubility limits of the materials used were pre-determined or known. Response surface methodology aims to investigate how changing certain input parameters in a process affects properties of interest in the product of that process [57]. Here we varied the amounts of quercetin, chitosan, and folic acid, expecting effects on the quercetin loading, surface charge, and folic acid content properties of the nanoparticles. These properties were measured and related mathematically to the input parameters using analysis of variance (ANOVA), which also identified which input variable or combination of variables produced statistically significant changes in the measurements within predefined confidence levels of 95% [58]. This mathematical relationship provided us with a model to predict what input values would maximize the quercetin loading, obtain the smallest positive surface charge, and maximize the folic acid content. The optimization process included a desirability function that was used to predict the combination of input values of 10 mg of quercetin, 23 mg of chitosan, and 45 mg of folic acid that would result in a product with the best possible response values of 7.02% quercetin loading, +2.52 mV surface charge, and 1.24% folic acid content. The experimental values matched the predicted values closely, indicating that the model was suitable. However, the R^2^ value of 0.85 for folic acid content (Figure S1C) indicates that 15% of the data is unexplained by the model and could suggest that this response was affected by a variable that was not accounted for in this study.

The design of experiments has been used successfully in the optimization of numerous polymeric nanoparticle systems using input parameters that had the most influence on the responses of interest. The most common measured responses have been mean particle size, polydispersity index, zeta potential, and entrapment efficiency [59,60,61]. In our study, we chose to maximize loading since quercetin is very well tolerated, with an LD50 of 160 mg/kg body weight [62]. It is more difficult to achieve high drug loading than high entrapment efficiency for most nano-systems [63], even though high drug loading has been associated with higher efficacy and better control over release properties [64]. Our focus on achieving maximum drug loading was also because this property has been found to be more significant in therapeutic effects and metabolism in in vivo studies [63,65]. We aimed for a small positive charge on the nanoparticles in order to promote attractive interactions with the negative cell membrane [66,67] and increase cellular penetration [62,63]. We also chose to maximize folic acid content in order to increase the likelihood of these moieties binding to the PSMA antigen on the cell surface [68,69,70,71], thereby enabling the nanoparticles to enter cancer cells more easily. 

Chu and co-workers [72] have described how nanofabrication techniques have hydrophobic drug loading limitations of approximately 10%, and a report by Lestari’s group [73] analyzed differently sized silica nanoparticles at drug loadings of 8.9% and 10%. They showed that there was a controlled, pH-dependent release of quercetin from the nanoparticle system, which is consistent with the results we observed. However, after 72 h, there was a maximum cumulative release of only 7% in acidic pH, indicating a much lower bioavailability than what we measured (65% at 72 h). Li and team [74] have reported on a pegylated nanoliposomal system with 8.5% drug loading that displayed a sustained release pattern with a much more favorable maximum release, while the similar sustained and maximum cumulative release was recorded by Davarnejad and co-workers [75] who used mixed nanomicelles with a relatively low drug loading of 2.3%. However, these systems were tested at pH 7.4, modeling the release in normal physiological pH, but no information can be extrapolated about the release in the characteristically acidic pH of the tumor microenvironment. Another study by Nan and team [15] describes quercetin-entrapped chitosan nanoparticles with a relatively high drug loading of 13.2% for topical applications. No drug release experiments were conducted in solution, but this work also highlighted the enhanced cellular uptake of the nanoparticles due to the chitosan component. As expected, the quercetin loading measurements for all the systems that we tested in Table 2 fall in the range of the above literature reports, while the charge, drug release, and biological activity of our final system (highlighted in bold) are compared with reports in Table 6.

Baksi and team [33] prepared quercetin-entrapped chitosan nanoparticles, which showed increased cytotoxicity on breast cancer cells compared to free quercetin. Interestingly, the in vitro kinetics data revealed that there were much higher percentages of drug release than we observed after 12 h (their final time point)—they reported 67% and 76% at pH 7.4 and 5.3, respectively, compared to our values of 35% and 38%, respectively, at pH values of 7.4 and 6.0, indicating a prolonged release by our system. Gupta’s group optimized folic acid-targeted PLGA nanoparticles for skin cancer [32], and as expected, they found similar release patterns for quercetin at pH 7.5 and 5.6. Yadav and co-workers [31] reported on PLGA nanoparticles that caused more destruction of cellular morphology and increased cytotoxicity on cervical and breast cancer cell lines when compared to free quercetin. We observed a similar trend regarding cytotoxicity in the PC-3 and, more especially, in the LnCap cell lines when comparing the non-targeted (uncoated) nano-system to free quercetin. 

Using chitosan, we formulated nanoparticles with a slightly positive surface charge of +1.84 mV, intended to minimize opsonization commonly found with highly charged nanoparticles [76], control release [77], and enhance cellular uptake [66]. However, in a study involving highly positive, slightly positive (+6 mV), and negatively charged SPIONS, an array of proteins preferentially bound to both the slightly and highly positive particles rather than the negatively charged particles [78]. Slightly more positive (+9 mV) chitosan nanoparticles were also prepared by [79] and investigated for their drug release of plasmid DNA. The study showed complete release in 24 h. More recently, Subramaniam and co-workers [80] aimed to fabricate chitosan nanoparticles with a charge between +1 and +10 mV for increased cellular penetration. The prepared nanoparticles were +19 mV, and it achieved 70% release of its payload after 4 h at pH 7.2. Several recent studies have demonstrated the controlled drug release behavior and enhanced cellular uptake and cytotoxicity of chitosan-based nanoparticles on cancer cells [77,81,82]. Even though the chitosan component in our optimized nano-system did show a controlled, pH-dependent release, we did not observe a significant increase in cellular uptake or cytotoxicity between the uncoated and coated nanoparticles on the PSMA-negative PC-3 cell line. This could be due to the fact that all the comparable studies have been performed on chitosan nanoparticles with a highly positive charge, which could promote their entry into the cancer cells where they could deliver their payload in a sustained manner and thereby cause increased cytotoxicity [67].

Our results also suggest that the increased cytotoxicity and cellular uptake observed in the PSMA-positive LnCap cell line is due to the presence of the folic acid component in the coated nanoparticles, which we maximized to 1.61%. Yao et al. [83] found that folic acid was able to inhibit the enzymatic functions of PSMA, suggesting competitive binding of folic acid to PSMA, and Hattori et al. [27] used folate-linked nanoparticles for DNA delivery to LnCap cells via the binding of PSMA, proposing folic acid as a ligand to target PSMA. Flores and co-workers [56] validated the binding and internalization of folic acid using a folate-conjugated fluorescently labeled probe. In agreement with the results we observed, they reported an increase in cytotoxicity and cellular uptake in the PSMA-positive LnCap cell line compared to the PSMA-negative PC-3 cell line when using a folic acid-conjugated nano-system. Jivrajani and co-workers [84] used folic acid-targeted bacterial minicells of siRNA delivery and showed a large degree of uptake using the folate-targeted minicells and no observable uptake of the non-targeted minicells, but no comparison was made on a cell line without folic acid binding receptors. Our results used LnCap and PC-3 cell lines, both of which have very low levels of folate receptors [27], and therefore, the difference in uptake can be equated to the level of PSMA expression in each cell line. There is also no significant difference in either the uptake or cytotoxicity between the uncoated (non-targeted) and coated (targeted) nano-system on the PC-3 cell line since this cell line has low levels of PSMA expression [27] and cannot selectively take up the targeted nano-system by PSMA binding. The inverse is true about the LnCap cell line, where high PSMA-specific cytotoxicity and cellular uptake are observed.

## 5. Conclusions

Our research presents the design, optimization, and evaluation of a folic acid-targeted nano-delivery system for prostate cancer. We have demonstrated that the optimized nano-system displayed a sustained, pH-dependent release profile and increased cancer cell uptake and toxicity when compared to free quercetin and the corresponding non-targeted system. This system shows potential as an actively targeted carrier for prostate cancer drug delivery. Future work should include the testing of this nano-system for particle stability over time, biocompatibility, and possibly another optimization process investigating the best surface charge for nanoparticles to simultaneously minimize opsonization and maximize cellular uptake.

## Figures and Tables

**Table 1:** Formulations generated by JMP software with varying amounts of quercetin (Q), chitosan (Chi), and folic acid (Fol).

Formulation	Parameters	Q/mg	Chi/mg	Fol/mg
P1	(1, 0, 0)	30	35	30
P2	(−1, −1, −1)	10	10	15
P3	(0, 0, −1)	20	35	10
P4	(1, 1, 1)	30	60	45
P5	(0, 0, 0)	20	35	30
P6	(0, 1, 0)	20	60	30
P7	(−1, 1, −1)	10	60	15
P8	(1, 1, −1)	30	60	15
P9	(1, −1, −1)	30	10	15
P10	(1, −1, 1)	30	10	45
P11	(0, −1, 0)	20	10	30
P12	(0, 0, 0)	20	35	30
P13	(−1, −1, 1)	10	10	45
P14	(0, 0, 1)	20	35	45
P15	(−1, 1, 1)	10	60	45
P16	(−1, 0, 0)	10	35	30

**Table 2:** Responses obtained using varying amounts of quercetin, chitosan, and folic acid, where QL = quercetin loading, Z potential = zeta potential, FA = folic acid content.

Formulation	Parameters	QL/%	Ζ Potential	FA/%
P1	(1, 0, 0)	9.04 ± 2.15	−1.60 ± 1.45	1.90 ± 0.15
P2	(−1, −1, −1)	3.01 ± 1.50	+2.33 ± 0.87	0.37 ± 0.15
P3	(0, 0, −1)	3.90 ± 1.05	+2.40 ± 0.08	0.77 ± 0.26
P4	(1, 1, 1)	8.27 ± 1.30	−5.03 ± 0.67	1.20 ± 0.25
P5	(0, 0, 0)	2.78 ± 1.71	+3.79 ± 1.21	0.44 ± 0.06
P6	(0, 1, 0)	1.83 ± 1.30	−2.17 ± 0.63	0.21 ± 0.17
P7	(−1, 1, −1)	1.71 ± 2.86	+3.83 ± 1.34	0.18 ± 0.15
P8	(1, 1, −1)	6.72 ± 1.30	−4.93 ± 0.17	0.66 ± 0.46
P9	(1, −1, −1)	8.94 ± 1.30	+1.07 ± 0.35	0.91 ± 0.10
P10	(1, −1, 1)	14.2 ± 2.39	−3.37 ± 0.31	1.48 ± 0,59
P11	(0, −1, 0)	5.18 ± 0.80	+1.58 ± 1.56	0.61 ± 0.21
P12	(0, 0, 0)	2.45 ± 1.67	+3.15 ± 0,47	0.54 ± 0.17
P13	(−1, −1, 1)	7.01 ± 1.05	−1.20 ± 0.61	0.95 ± 0.25
P14	(0, 0, 1)	6.12 ± 1.30	+2.39 ± 0.85	1.13 ± 0.21
P15	(−1, 1, 1)	6.05 ± 0.80	+3.28 ± 0.36	0.95 ± 0.15
P16	(−1, 0, 0)	3.42 ± 1.30	+2.26 ± 0.29	0.70 ± 0.25

**Table 3:** Responses obtained using varying amounts of quercetin, chitosan, and folic acid.

Response	Predicted	Actual	Bias
Quercetin Loading	7.02	7.11 ± 1.60	+0.0126
Surface Charge	+2.52	+1.84 ± 0.40	−0.370
Folic Acid Content	1.24	1.61 ± 0.35	+0.236

**Table 4:** Summary of dynamic light scattering measurements of uncoated and coated nanoparticles.

Formulation	Size/nm	PDI	ζ Potential/mV
Uncoated nps	159.8 ± 2.0	0.068 ± 0.01	−21.0 ± 1.6
Coated nps	206.2 ± 1.7	0.069 ± 0.002	+1.84 ± 0.4

**Table 5:** Fluorescent intensities of LnCap and PC-3 cells treated with coated and uncoated nanoparticles.

Cell Line	Treatment	% Fluorescence
LnCap	Coated nps	2.79 ± 0.50
PC-3	Uncoated nps	1.86 ± 0.22
	Coated nps	1.72 ± 0.37
	Uncoated nps	1.99 ± 0.39

**Table 6:** Comparison of fabricated PLGA-chitosan folic acid system with previously reported results.

System	ζ Potential/mV	Drug Release	Biological Activity
Silica nanoparticles	Varied	7% after 72 h	Not specified
Pegylated quercetin liposomes	−13.1	85% after 96 h	Increased cytotoxicity in cervical cancer cells and greater reduction in tumor size in a mouse model compared to free quercetin
Nanomicelles	Not specified	83.6% after 120 h	Increased cytotoxicity on breast cancer cells compared to free quercetin
Chitosan-quercetin nps	+22.53	76% after 12 h	Increased cytotoxicity on lung and breast cancer cells and greater reduction in tumor volume in a mouse model compared to free quercetin
SPIONS	Varied	Not applicable	Increased protein binding of positively charged particles
Chitosan nps	+9	100% after 24 h	Not specified
Folate-linked nps	+19	70% after 4 h	PSMA binding on LnCap cells
Folic acid-conjugated nps	Not specified	90% after 24 h	Increased cytotoxicity of therapeutic peptide and greater cellular uptake in LnCap cells
Folic acid minicells	Not specified	Not applicable	Increased cellular uptake in LnCap cells
**PLGA-quercetin chitosan folic acid nps**	**+1.84**	**78% after 168 h**	**Increased cytotoxicity of quercetin and greater cellular uptake in LnCap cells**

## Data Availability

Additional available data can be obtained from the authors upon request.

## Supplementary Materials

The following supporting information can be downloaded at: https://www.mdpi.com/article/10.3390/biomedicines11041201/s1, Figure S1: Actual vs. predicted plots of quercetin loading (**A**), charge (**B**), and folic acid content (**C**)—from JMP V17; Figure S2: Residual plots of quercetin loading (**A**), charge (**B**) and folic acid content (**C**)—from JMP V17; Figure S3: Main effects data obtained using a central composite design—from JMP V17; Figure S4: 1–2: Representative graphs of size and potential of **A**—uncoated nanoparticles and **B**—coated nanoparticles. Figure S5: Cell viability of 3T3-NIH cells after being treated with unloaded uncoated (**A**) and coated (**B**) nanoparticles for 48 and 72 h.
