# Supplementary material for: Design of Chitosan-Coated, Quercetin-Loaded PLGA Nanoparticles for Enhanced PSMA-Specific Activity on LnCap Prostate Cancer Cells
Source: Biomedicines. 2023 Apr 18;11(4):1201. doi: 10.3390/biomedicines11041201 (PMC10136298; doi:10.3390/biomedicines11041201)
Supplement: Supplementary file 1 [file biomedicines-11-01201-s001.zip › biomedicines-2283626-supplementary.pdf]

biomedicines

MDPI

Supplementary Material

Design of Chitosan-Coated, Quercetin-Loaded PLGA Nanoparticles for Enhanced PSMA-Specific Activity on LnCap Prostate Cancer Cells

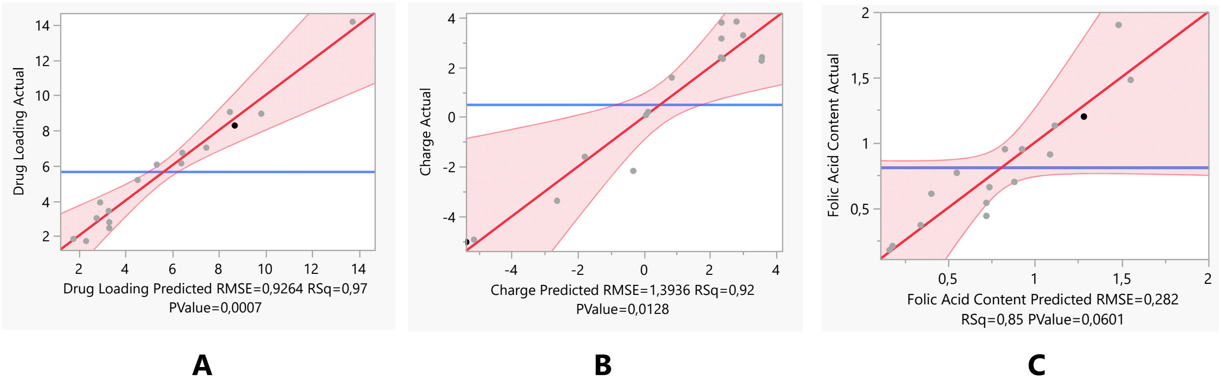

Figure S1. Actual vs Predicted plots of quercetin loading **(A)**, charge **(B)** and folic acid content (**C**) – from JMP V17

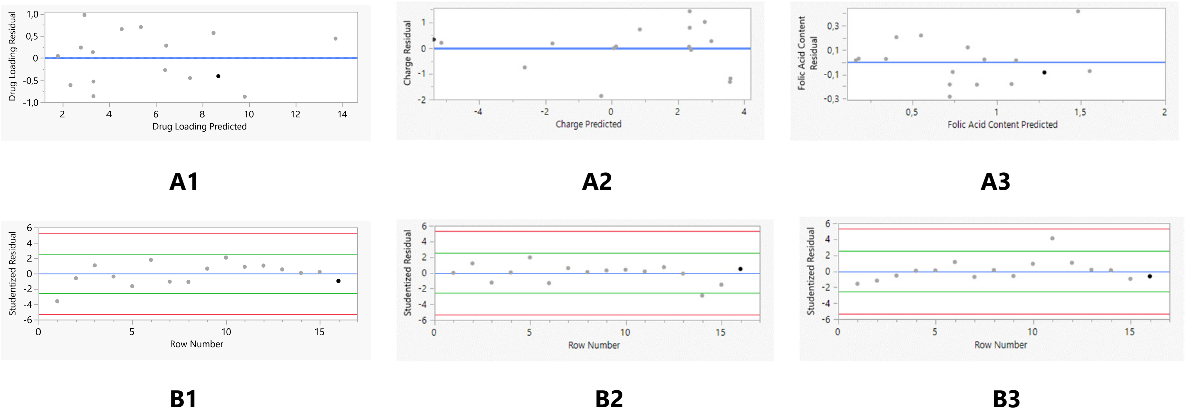

Figure S2. Residual Predicted plots (**A**) and Studentized Residuals **(B)** of quercetin loading (**1**), charge (**2**) and folic acid content (**3**) - from JMP V17

Source	Logworth	PValue
Quercetin(10,30)	3,937	0,00012
Folic Acid(15,45)	2,983	0,00104
Chitosan(10,60)	2,470	0,00338
Quercetin*Quercetin	2,387	0,00410
Quercetin*Chitosan	1,536	0,02912
Chitosan*Chitosan	1,313	0,04861
Folic Acid*Folic Acid	1,251	0,05614
Chitosan*Folic Acid	0,601	0,25083
Quercetin*Folic Acid	0,243	0,57098

Figure S3. Main effects data obtained using a central composite design – from JMP V17

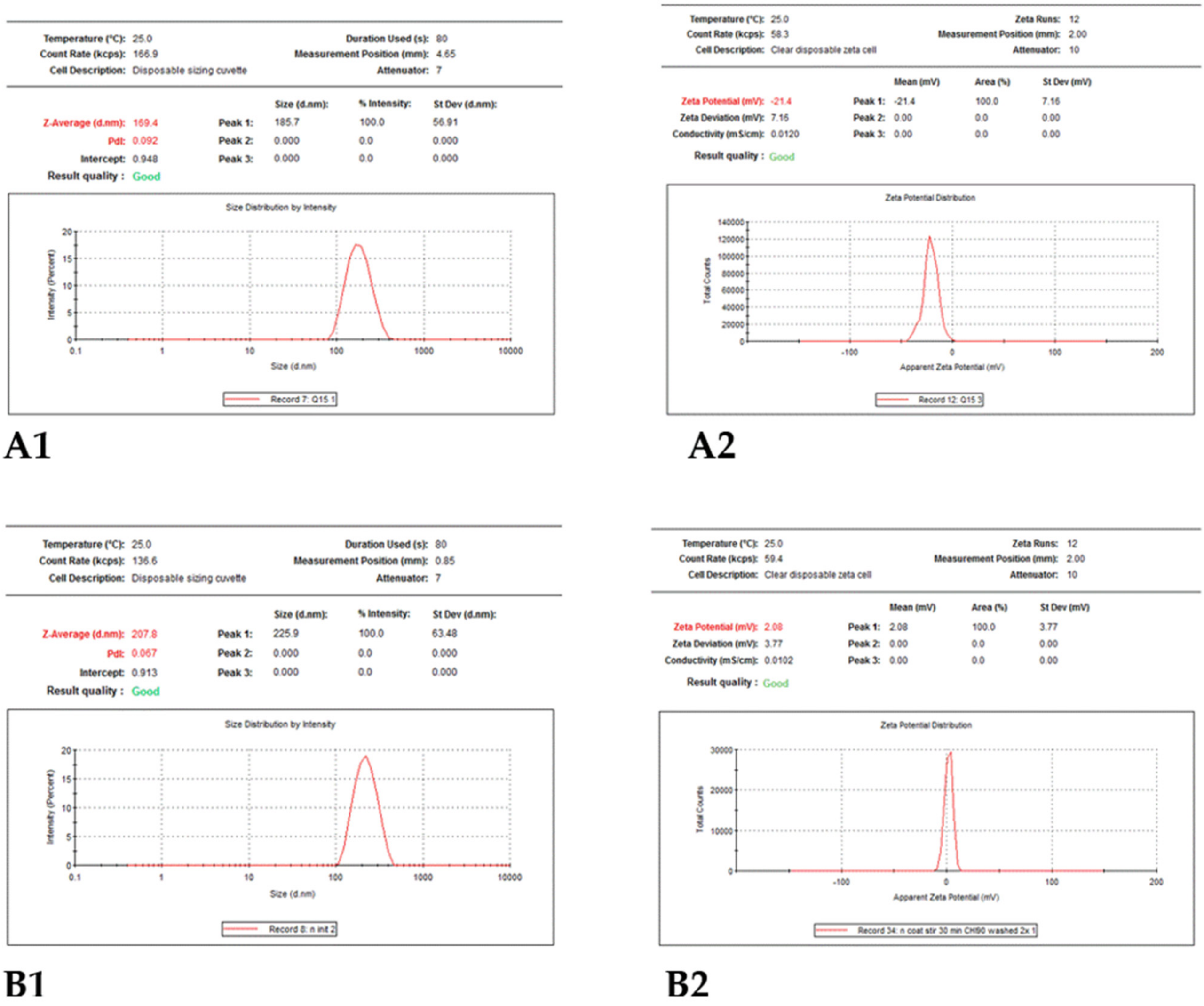

Figure S4. 1-2: Representative Graphs of Size and Potential of **A**- un-coated nanoparticles and **B**- coated nanoparticles.

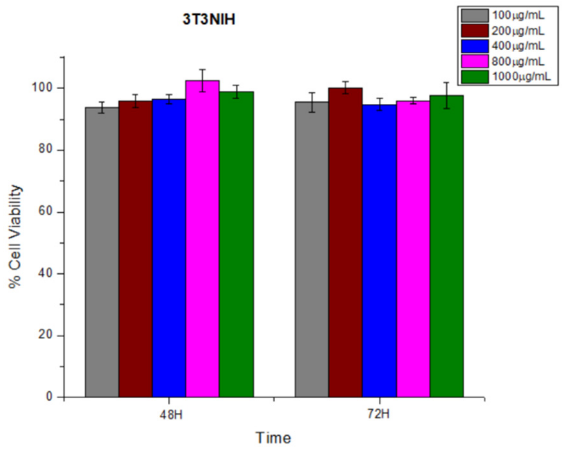

A

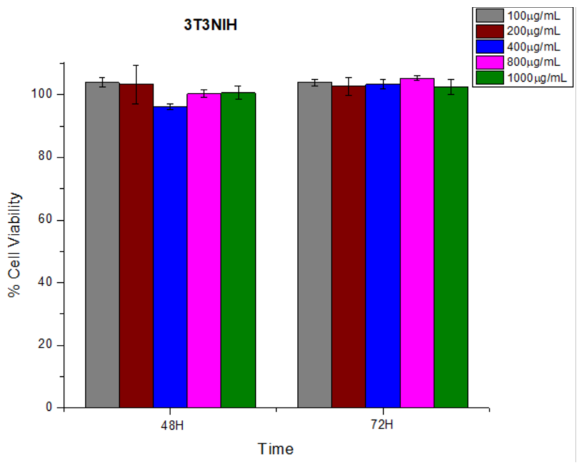

B

Figure S5. Cell viability of 3T3-NIH cells after being treated with unloaded uncoated (**A**) and coated (**B**) nanoparticles for 48 and 72 hours
